# Clinical Notes

**Published:** 1888-07

**Authors:** Elizabeth W. M. Cameron

**Affiliations:** New York


### CLINICAL NOTES.

I. Miss Addie S., age twenty-three, of Harlem, has had for past three years an intense craving for salt; covered her food with it and ate it by the spoonful several times a day. Also used tea excessively, and for years had been in the habit of taking Quinine for every indisposition, colds, etc.

I saw her first about February 24th (seven P. M.), and gave her one dose of Dr. Fincke's Natrum muriaticum $^{45m}$. Saw her again on the 25th about four P. M., and she said that she had not wanted any salt for breakfast nor for lunch, and up to this time her craving for salt had not returned.

A week later was told that she had taken a dose of Quinine for a cold, and it made he very sick at her stomach. She has attempted to take it since but with like results, and can no longer use it.

Four weeks after taking the Natr. mur. she had lost all craving for tea, and no longer drinks *any*, while she was accustomed to drink three and four cups at every meal.

I gave but one dose, and if I am not mistaken, it was the only dose of homœopathic medicine she had ever taken. The cure has been permanent.

II. Mrs. N. A. G., age thirty, Harlem, received a shock last October on being told that she was liable to arrest if she sold the mother tincture of Aconite without a physician's prescription. Since then she has suffered from excessive nervousness and constant dread. Had to have some one stay with her all the time.

I saw her for the first time on February 21st. Gave her one dose of Sulphur$^{200}$, and left one dose of Fincke's Natrum muriaticum$^{45m}$ for her to take four days later. From that time on she took no other remedy.

About a month later I received a message from her stating that she had entirely recovered. I had not seen her in the meantime. Also received following note:

"NEW YORK, April 3d, 1888.

"DEAR DOCTOR:

"The medicine you prescribed for me has worked a complete cure. I am now feeling perfectly free from the nervous trouble, and am well and getting stout. Many thanks to you for your kindness. "Very gratefully,

"N. A. G."

III. Last fall X. proved a remedy (the name of which he cannot recall at present) which had special action on the arterial circulation. It left him with marked strabismus of both eyes, and very marked ascites and dyspnœa on slight exertion. After trying several remedies without any change, he took one dose of Theridion$^{200}$. It caused a great discharge of water, per rectum, which lasted for about two days and then disappeared. The strabismus also disappeared.

Seven days later X. took a second dose, when the discharge came on again. This time very frequent, in large quantities, and very bad smelling. Had very little pain, slight nausea, very dry tongue. This attack lasted about two days, when the dyspnœa was greatly improved, and the ascites had entirely disappeared, leaving the abdoman perfectly flat.

About ten days afterward took another dose, and three or four days later had a slight fever, followed by a violent, shaking chill, which lasted a day; felt as though every bone in his body would break. Mouth *very dry*, headache in left temple and over left eye. In fact, X. got an excellent proving of the drug from this third dose. Was in bed two days with this attack, and was left *very* weak for some days.

After a few days there appeared around the umbilicus, mostly on the left side, a bright red eruption which itched violently, and a similar patch in each lumbar region and at the *left* ant. sup. spinous process of the ilium. There was a shining, sticky discharge from the eruption at the umbilicus, which gave the patch a varnished appearance. There also seemed a slight moisture from the umbilicus. Also had the profuse, thick, yellow, bad-smelling discharge from the nose as described under Theridion. After this discharge had lasted for two weeks, X.

took Apis.$^{1m}$, and after taking a few doses the eruption and discharge entirely disappeared.

Following the chill there was an intense aching in the bones of the feet and soreness of the soles, also an acute attack of piles, which disappeared after the first dose of Apis.

ELIZABETH W. M. CAMERON, M. D.

NEW YORK.

